# The evolution of sex peptide: sexual conflict, cooperation, and coevolution

**DOI:** 10.1111/brv.12849

**Published:** 2022-03-06

**Authors:** Ben R. Hopkins, Jennifer C. Perry

**Affiliations:** ^1^ Department of Evolution and Ecology University of California – Davis One Shields Avenue Davis CA 95616 U.S.A.; ^2^ School of Biological Sciences University of East Anglia Norwich NR4 7TJ U.K.

**Keywords:** sperm competition, sex peptide, signalling, condition dependence, sexual conflict, sexual selection, seminal fluid, ejaculates, post‐mating responses, coevolution

## Abstract

A central paradigm in evolutionary biology is that the fundamental divergence in the fitness interests of the sexes (‘sexual conflict’) can lead to both the evolution of sex‐specific traits that reduce fitness for individuals of the opposite sex, and sexually antagonistic coevolution between the sexes. However, clear examples of traits that evolved in this way – where a single trait in one sex demonstrably depresses the fitness of members of the opposite sex, resulting in antagonistic coevolution – are rare. The *Drosophila* seminal protein ‘sex peptide’ (SP) is perhaps the most widely cited example of a trait that appears to harm females while benefitting males. Transferred in the ejaculate by males during mating, SP triggers profound and wide‐ranging changes in female behaviour and physiology. Early studies reported that the transfer of SP enhances male fitness while depressing female fitness, providing the foundations for the widespread view that SP has evolved to manipulate females for male benefit. Here, we argue that this view is (*i*) a simplification of a wider body of contradictory empirical research, (*ii*) narrow with respect to theory describing the origin and maintenance of sexually selected traits, and (*iii*) hard to reconcile with what we know of the evolutionary history of SP's effects on females. We begin by charting the history of thought regarding SP, both at proximate (its production, function, and mechanism of action) and ultimate (its fitness consequences and evolutionary history) levels, reviewing how studies of SP were central to the development of the field of sexual conflict. We describe a prevailing paradigm for SP's evolution: that SP originated and continues to evolve to manipulate females for male benefit. In contrast to this view, we argue on three grounds that the weight of evidence does not support the view that receipt of SP decreases female fitness: (*i*) results from studies of SP's impact on female fitness are mixed and more often neutral or positive, with fitness costs emerging only under nutritional extremes; (*ii*) whether costs from SP are appreciable in wild‐living populations remains untested; and (*iii*) recently described confounds in genetic manipulations of SP raise the possibility that measures of the costs and benefits of SP have been distorted. Beyond SP's fitness effects, comparative and genetic data are also difficult to square with the idea that females suffer fitness costs from SP. Instead, these data – from functional and evolutionary genetics and the neural circuitry of female responses to SP – suggest an evolutionary history involving the evolution of a dedicated SP‐sensing apparatus in the female reproductive tract that is likely to have evolved because it benefits females, rather than harms them. We end by exploring theory and evidence that SP benefits females by functioning as a signal of male quality or of sperm receipt and storage (or both). The expanded view of the evolution of SP that we outline recognises the context‐dependent and fluctuating roles played by both cooperative and antagonistic selection in the origin and maintenance of reproductive traits.

## I INTRODUCTION

Sexual evolution and coevolution walk a fine line between antagonistic and cooperative selection. On the one hand, some cooperation is required to unite sperm and egg. On the other, non‐clonally related individuals have distinct and divergent fitness interests, such that sexual interactions that maximise female fitness may not maximise male fitness (and *vice versa*) (Parker, 1979). How great a mark this conflict of interests leaves on the design and evolution of each sex has been one of the most hotly debated topics in evolutionary biology in recent decades (Eberhard, 1996, 2009; Holland & Rice, 1997, 1998; Rice & Holland, 1997; Chapman *et al*., 2003a; Cordero & Eberhard, 2003, 2005; Pizzari & Snook, 2003, 2004; Arnqvist, 2004; Arnqvist & Rowe, 2005; Tregenza, Wedell & Chapman, 2006; Rice & Gavrilets, 2014; Perry & Rowe, 2015). Yet, several decades on from the emergence of the field of sexual conflict, robust empirical demonstrations of single traits that enhance the fitness of one sex at the expense of the other – especially traits for which fitness effects are quantifiable and underlying loci identified – remain few and far between (Perry & Rowe, 2018; Rowe, Chenoweth & Agrawal, 2018). Even scarcer are examples of the coevolution of antagonistic traits between the sexes.

**Fig. 1:**
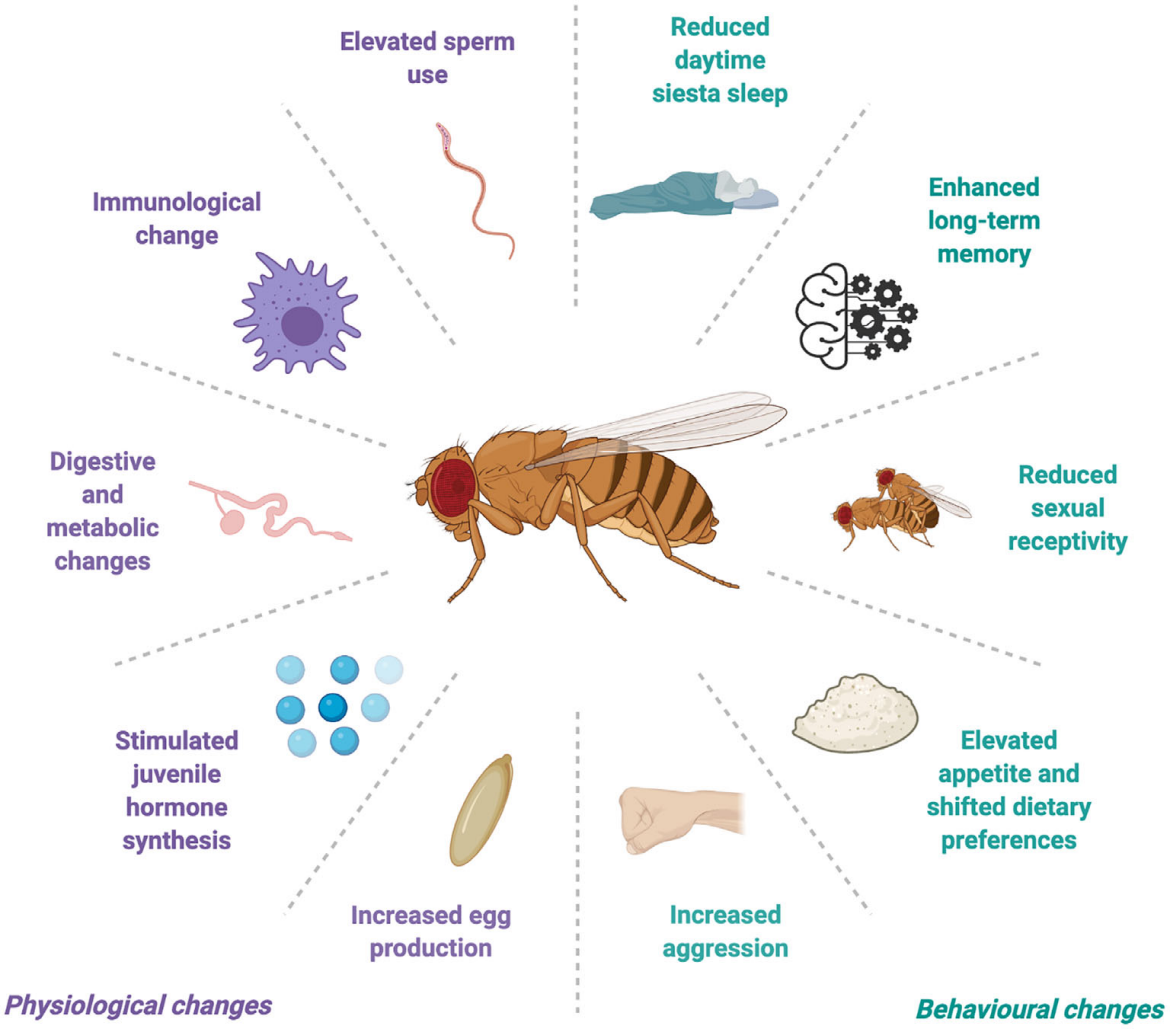
A wide variety of behavioural and physiological changes take place in females upon receipt of sex peptide (SP), including shifts in feeding (Carvalho *et al*., 2006; Ribeiro & Dickson, 2010; Walker *et al*., 2015), memory (Scheunemann *et al*., 2019), sleep and movement (Isaac *et al*., 2010), aggression (Bath *et al*., 2017), sexual receptivity and egg‐laying (Chen *et al*., 1988; Aigaki *et al*., 1991; Chapman *et al*., 2003b; Liu & Kubli, 2003), gut activity (Cognigni *et al*., 2011; Reiff *et al*., 2015; White *et al*., 2021), sperm use (Avila *et al*., 2010), immune activity (Peng *et al*., 2005b; Domanitskaya *et al*., 2007; Schwenke & Lazzaro, 2017), and, presumably underlying much of this, changes in gene expression (Gioti *et al*., 2012) and endocrine activity (Soller *et al*., 1999). Figure adapted from the Hallmarks of Cancer: Circle template, by BioRender.com. Retrieved from https://app.biorender.com/biorender‐templates.

Seminal fluid, a rapidly evolving molecular cocktail transferred to females alongside sperm, has been a focal point for research on male–female conflict and coevolution (e.g. Chapman *et al*., 1995, 2003a, 2003b; Rice, 1996; Stockley, 1997; Holland & Rice, 1998; Civetta & Clark, 2000; Swanson & Vacquier, 2002; Perry, Sirot & Wigby, 2013; Sirot *et al*., 2015; Wilburn & Swanson, 2016; Smith *et al*., 2017). One seminal fluid component, the sex peptide (SP) of the fruit fly *Drosophila melanogaster*, has played a foundational role in our understanding of the evolution of not only seminal fluid, but of reproductive traits more broadly. Just 36 amino acids long in its mature form, this male‐specific protein induces profound and long‐lasting changes in female physiology and behaviour (Chen *et al*., 1988) (Fig. 1). The breadth and nature of these changes raises a fundamental question: why do males exert such influence over female reproductive processes? The paradigmatic view is that SP's influence evolved *via* sexually antagonistic selection: males use SP to manipulate female reproductive processes to their gain and at a cost to the female. This paradigm has developed over recent decades from a set of intersecting research streams, including sexual conflict theory (Parker, 1979; Holland & Rice, 1998), empirical studies of mating costs (Fowler & Partridge, 1989; Chapman, Hutchings & Partridge, 1993; Chapman *et al*., 1995; Civetta & Clark, 2000; Wigby & Chapman, 2005), and mechanistic studies of the pathways through which SP acts in females (Yapici *et al*., 2008; Kim *et al*., 2010; Brockhurst *et al*., 2014; Tsuda *et al*., 2015). SP is now widely viewed as a ‘poster child’ for sexual conflict: a single trait, pinpointed to a single locus, thought to have evolved to promote male reproductive success at female expense. It might be the most widely cited example of such a trait – certainly one of the best studied – and it is regularly placed front and centre in primers on sexual conflict (e.g. Fricke *et al*., 2009a; Westneat & Fox, 2010; Wyatt, 2014; Shuker & Simmons, 2014; Hosken, Archer & Mank, 2019).

Here, we argue that this conflict paradigm for SP is inconsistent with most direct studies of the effects of SP on female fitness; narrow with respect to theory describing the origin and maintenance of sexually selected traits; assumes costs that are in fact rarely demonstrated, unknown in natural populations, and possibly confounded by artefacts of genetic manipulations; and hard to reconcile with what we know of the evolutionary history of the effects of SP on females. We begin by charting the historical development of the conflict paradigm for SP, before laying out a set of challenges to it. First, empirical studies of the fitness effects of SP receipt have offered mixed results: only one reports fitness costs for females (Wigby & Chapman, 2005) and another, which did not measure offspring production, reports only decreased female lifespan (Tower *et al*., 2017). Others have found neutral or even positive effects (Rogina, 2009; Fricke, Bretman & Chapman, 2010; Wensing & Fricke, 2018). Crucially, costs and neutral effects appear restricted to specific contexts, such as when females experience nutritional extremes (Wigby & Chapman, 2005; Rogina, 2009; Fricke *et al*., 2010). Second, whether costs from SP are appreciable in wild‐living populations remains untested. Finally, the recently described role for SP in mediating the transfer of other seminal fluid proteins (Wainwright *et al*., 2021) raises the possibility that genetic manipulations of SP may be confounded, distorting measurements of the fitness effects it has on females. After reviewing these challenges, we describe alternative scenarios for the origin and maintenance of SP and its interactions with females, emphasising the fluctuating roles played by both cooperative and antagonistic selection. By comparing relevant literature on the function and evolutionary genetics of SP and its receptor (SPR), and the neural circuitry underlying the female post‐mating response, we argue that a pure conflict model is poorly supported by the data. Instead, females might benefit from the receipt of SP as a signal of sperm receipt and retention in storage, a long‐standing idea (e.g. Manning, 1967), or as a signal of male quality (or both). We explore this possibility and discuss what this system can teach us about the evolution of signals and male–female interactions.

## II SEXUAL CONFLICT AND SEX PEPTIDE: THE DEVELOPMENT OF A PARADIGM

### (1) A brief history of sexual conflict

Traditional sexual selection models describe the evolution of female preference through the direct (e.g. parental care) or indirect (e.g. ‘good genes’) benefits that females accrue from mating with preferred males, resulting in the coevolution of female preferences and preferred male traits (Andersson, 1994). These ideas are at the heart of sexual signalling theory, which describes how ‘handicap’ and condition‐dependent signals can act as conspicuous advertisements of male quality, which females gain by attending to when selecting a mate (Zahavi, 1975; Grafen, 1990). Females can also benefit from mate preferences exerted after mating *via* sperm competition (when high‐quality males prevail) and cryptic female choice (Parker, 1970; Thornhill, 1983; Eberhard, 1996; Lüpold *et al*., 2020), and *via* ‘differential allocation’, wherein individuals plastically alter their reproductive investment in relation to their partner's traits [e.g. quality (Burley, 1988; Sheldon, 2000)].

The development of sexual conflict theory marked a paradigm shift in the way researchers thought about the evolution of sexual traits. The long‐held view that mating was largely a harmonious venture was subsumed by the recognition that, unless mating partners are clonally related, their evolutionary interests diverge (Parker, 1979, 2006; Chapman *et al*., 2003a; Arnqvist & Rowe, 2005; Tregenza *et al*., 2006). The nature of the sex roles – themselves a downstream product of anisogamy – is that for males, fitness rises more steeply with mating number than it does for females (Darwin, 1871; Bateman, 1948; Andersson, 1994; Dewsbury, 2005; Parker, 2014; Janicke *et al*., 2016). Consequently, for traits that result from sexual interactions – such as mating and parental care – the sexes cannot escape each other to reach a sex‐specific optimum. With sexually divergent routes to fitness maximisation, sex‐specific traits that maximise the evolutionary interests of their bearers may fail to maximise or even be antagonistic to the fitness interests of members of the other sex (Parker, 1979). This perspective raised the possibility that males and females could become locked in ‘chase‐away’ coevolutionary dynamics, in which traits that evolve in one sex to bring the value of a shared trait (such as mating rate) closer to that sex's optimum are met with counter‐adaptations in the other sex that shift the shared trait towards that sex's optimum instead (Rice & Holland, 1997; Holland & Rice, 1998; Gavrilets, 2000; Gavrilets, Arnqvist & Friberg, 2001; Parker, 2006). Such coevolution might proceed in any of several ways: for example, through an escalating ‘arms race’ involving the exaggeration of armaments, through cyclical ‘Red Queen’ dynamics, or through the evolution of female insensitivity to male antagonistic traits, rendering male traits ineffective (Parker, 1979; Gavrilets *et al*., 2001; Rowe, Cameron & Day, 2005; reviewed by Arnqvist & Rowe, 2005).

Sexual conflict and the sexually antagonistic coevolution it can initiate are now widely recognised as potent and ubiquitous drivers of evolutionary change (reviewed by Arnqvist & Rowe, 2005; Rice & Gavrilets, 2014). But despite the centrality of sexual conflict to contemporary evolutionary thought, and discussion of sexual conflict in major early works (e.g. Williams, 1966; Parker, 1970; Trivers, 1972), it was not until the mid‐1990s that interest surged (Arnqvist & Rowe, 2005; Parker, 2014). The roots of this resurgence lay in several key research streams. One concerned the mating systems of water striders, where sex‐specific costs and benefits of mating appear to have initiated a sexually antagonistic arms race in which males have evolved elaborate structures for grasping resistant females and females have evolved corresponding structures that reduce the effectiveness of male claspers (Rowe *et al*., 1994; Arnqvist & Rowe, 2002). Another was the study of ‘sensory exploitation’, where preferences for traits, such as mating calls or ornaments, pre‐date the traits themselves (Ryan, 1990). This idea predicted that male traits could evolve to ‘exploit’ sensory predispositions in females and manipulate their decision‐making. Sensory exploitation provided a backbone to Holland and Rice's ‘chase‐away’ model: female costs from sensory exploitation could drive sexually antagonistic coevolution (Holland & Rice, 1998).

Another research stream that pushed sexual conflict to the forefront of evolutionary biologists' minds in the mid‐1990s was the discovery that *D. melanogaster* females experience survival costs from exposure to males (Partridge *et al*., 1986). First came the discovery that mating itself, independent of egg production, reduces female lifespan (Fowler & Partridge, 1989). Then came the finding that mating costs are not driven by the receipt of sperm (Chapman *et al*., 1993), closely followed by the discovery that seminal fluid products (produced in the ‘main cells’ of the accessory glands) underlay female mating costs, and that the greater the exposure of females to these products, the greater the cost (Chapman *et al*., 1995; see also Kuijper, Stewart & Rice, 2006). These seminal products were already known to perform functions with clear benefits to males, including reducing female receptivity to remating and stimulating female egg‐laying (Manning, 1962, 1967; David, 1963; Chen, 1984; Chen *et al*., 1988; Aigaki *et al*., 1991). Here, then, was an apparent sexual conflict. The findings from fruit flies paralleled similar findings from *Caenorhabditis elegans* that were reported around the same time (Gems & Riddle, 1996). Holland & Rice (1998, p. 5) used both examples to assert that in these species ‘seminal fluid proteins are toxic to females, such that the more they receive the more harm is done’.

The notion of seminal fluid toxicity continued to grow in the late 1990s and early 2000s. Experimental evolution in *D. melanogaster* showed that if females were prevented from coevolving with males, then males evolved increased fitness at the expense of their mates (Rice, 1996). The fitness gains for males, and the costs for their female partners, were associated with seminal fluid‐mediated traits: an increased ability to limit their partner's remating and to resist sperm displacement by rival males. Three years later, Holland & Rice (1999) showed that under enforced monogamy, which aligns the fitness interests of males and females, males evolve to become less harmful to females and females become less resistant to male‐induced harm. Theory followed that suggested that the transfer of ‘toxins’ that inhibit mating could be a stable and general male adaptation to sperm competition (Johnstone & Keller, 2000), while experimental work revealed a positive genetic correlation between male performance in sperm competition and post‐mating female mortality (Civetta & Clark, 2000).

Collectively, these studies set the evolution of seminal fluid within sexual conflict and antagonistic coevolution: some seminal products appear to evolve to give males an advantage in sperm competition, but at a cost to the fitness of their partners, who evolve resistance in response. This framework provided a potential explanation (e.g. Rice & Holland, 1997; Parker & Partridge, 1998; Gavrilets, 2000; Swanson & Vacquier, 2002) for the rapid evolution of some seminal fluid proteins (SFPs) reported around the same time (Aguade, Miyashita & Langley, 1992; Tsaur & Wu, 1997; Tsaur, Ting & Wu, 1998).

As the sexually antagonistic view of sexual interactions, and SFPs in particular, gained popularity, some authors voiced concerns (reviewed by Hosken & Stockley, 2004; Parker, 2006, 2014). The first was that measurements of costs and benefits under laboratory conditions might bear little relevance to those experienced during a species’ evolutionary history (Cordero & Eberhard, 2003; Eberhard *et al*., 2003; Eberhard, 2009). The second related to the bookkeeping of fitness effects: the direct costs incurred by a female from male manipulation must be weighed against the benefits she could accrue *via* her offspring (Cordero & Eberhard, 2003; Pizzari & Snook, 2003). Direct costs imposed by SFPs (or other male traits) might be offset and even outweighed by direct and indirect benefits. Females might gain directly by using the receipt of seminal products as a cue to initiate reproductive processes (Eberhard, 1996). They might also gain by expressing a threshold for responses to seminal substances, thereby ensuring that only males transferring qualitatively or quantitatively ‘superior’ ejaculates can trigger maximum responses (Cordero, 1995; Eberhard & Cordero, 1995; Eberhard, 1996). In this way, females could gain indirect benefits through either improved offspring viability, if these seminal qualities reflect a male's ‘good genes’, or the production of sons that are better able to stimulate their own mates (the ‘sexy sons’ effect; Weatherhead & Robertson, 1979).

Despite the empirical challenges of distinguishing sexual conflict and indirect benefit explanations for apparently harmful traits (Cordero & Eberhard, 2003; Eberhard, 2009, 2010, 2015; Eberhard & Lehmann, 2019), the scope for indirect benefits to offset direct costs from harmful partners is expected to be limited because indirect benefits are necessarily diluted by the strength of genetic covariance between male harmful traits and female responses to those traits (Kirkpatrick & Barton, 1997; Cameron, Day & Rowe, 2003) (see also Chapman *et al*., 2003a; Orteiza, Linder & Rice, 2005; Parker, 2006). By 2006, the general consensus was that while indirect benefits surely exist, it is unlikely that they would offset direct costs to females (Tregenza *et al*., 2006).

### (2) The biology of sex peptide

While the field of sexual conflict was developing, so too was understanding of the functional biology of seminal fluid. In the late 1950s, a male‐specific peptide produced by the accessory glands, termed a ‘sex peptide’, was identified in *D. melanogaster* (Fox, Mead & Munyon, 1959; Chen & Diem, 1961). But it was not until decades later that post‐mating responses in females, specifically elevated ovulation and oviposition and reduced sexual receptivity, were unambiguously shown to be induced by SP, by injection of the purified peptide into the female abdomen (Chen *et al*., 1988). Several complementary approaches have since confirmed that these effects are attributable to SP: ectopic SP expression in virgin females (Aigaki *et al*., 1991), knockout of the SP gene (Liu & Kubli, 2003), and RNA interference (RNAi)‐mediated knockdown of SP expression (Chapman *et al*., 2003b).

Since its characterisation, considerable progress has been made in understanding what SP does and how it does it. The list of post‐mating phenotypes that SP mediates has grown far longer than stimulating fecundity and reducing receptivity (Fig. 1). The effects of SP are so varied and dramatic that authors routinely describe SP as flicking a ‘switch’ between virgin and mated states (Yapici *et al*., 2008; Kubli & Bopp, 2012; Hussain *et al*., 2016). SP binds at its N‐terminus to the surface of sperm, from which the C‐terminus – which induces female post‐mating responses (Schmidt *et al*., 1993a) – is gradually cleaved at a trypsin cleavage site while sperm are in the female storage organs (Peng *et al*., 2005a). The binding of SP to sperm relies on a network of SFP co‐factors, and it is through SP's association with sperm that the duration of the post‐mating response is extended (‘the sperm effect’; Manning, 1967): from the ~1 day observed upon injection of SP into the female to upwards of 10 days in natural matings (Peng *et al*., 2005a; Ravi Ram & Wolfner, 2007, 2009; Gligorov *et al*., 2013; Findlay *et al*., 2014; Sitnik *et al*., 2016; Singh *et al*., 2018). SP brings about many behavioural and physiological changes in females by binding to SPR (Yapici *et al*., 2008). It is SPR's specific expression within a small number of internal sensory neurons (‘SP‐sensing neurons’) in the female reproductive tract that is required for the induction of post‐mating responses (Yapici *et al*., 2008; Häsemeyer *et al*., 2009; Rezával *et al*., 2012; Feng *et al*., 2014).

### (3) Sex peptide's place in the field of sexual conflict

By the end of 2003, two things appeared clear: (*i*) seminal fluid was the basis of the costs of mating incurred by females; and (*ii*) SP triggered the core female post‐mating responses. It was at this time that a gene‐knockdown line for *Sex Peptide* became available (Chapman *et al*., 2003b), allowing the connection between SP and mating costs to be directly tested. Wigby & Chapman (2005) demonstrated that SP alone was a major contributor to the cost of mating. Their elegant work showed that females housed with SP‐transferring males experience lower lifetime egg and offspring production compared with females housed with mutant knockdown males that do not transfer SP. Additionally, experimental females that did not receive SP lived at least as long as controls, despite mating 12‐fold more often (on account of their abnormally high receptivity). *Sex Peptide* was therefore the first gene shown likely to “play a central role in sexual conflict” (Wigby & Chapman, 2005, p. 316).

The significance of this result was quickly recognised. References to SP as a ‘harmful’ or ‘toxic’ trait (Lessells, 2006; Taylor, Wedell & Hosken, 2008; Brown *et al*., 2009; Hollis, Fierst & Houle, 2009; Cayetano *et al*., 2011; Rankin, Dieckmann & Kokko, 2011; Slatyer *et al*., 2012; Bonduriansky, 2014; Brooks & Garratt, 2017; Garcia‐Roa *et al*., 2020); ‘reducing’, ‘depressing’, ‘lowering’ or having a ‘negative’, ‘deleterious’, or ‘detrimental’ effect on female fitness (Andrés *et al*., 2006; Pröschel, Zhang & Parsch, 2006; Vahed, 2007; Barnes *et al*., 2008; Hosken *et al*., 2009; Hotzy & Arnqvist, 2009; Karl & Fischer, 2013; Weber, Patlar & Ramm, 2020); coming at a ‘cost to female lifetime reproductive success’ (Holman & Kokko, 2013); imposing ‘net fitness costs’ on females (Alonzo & Pizzari, 2010; Pizzari & Gardner, 2012); or being used to ‘exploit’ or ‘manipulate’ them (Bussiégre *et al*., 2006; Fischer, 2007; Reumer, Kraaijeveld & van Alphen, 2007; Hall *et al*., 2010; Price *et al*., 2010; Adler & Bonduriansky, 2014; Edward, Stockley & Hosken, 2015; Pizzari, Biernaskie & Carazo, 2015; Nallasivan *et al*., 2021) have since become widespread. Here was a clear example of a single male trait, identifiable at the level of a single locus and protein, that caused measurable fitness depression in female mates, and where the male trait targeted a female trait that was also identifiable at locus level. It is hard to overstate the importance of this case study in a field in which many male traits were thought to harm females, but where there were few cases in which harm to fitness could be quantified and vanishingly few where a corresponding female trait could be identified (Arnqvist & Rowe, 2005; Perry & Rowe, 2015). For these reasons, the example of SP and its sexually antagonistic effects is now used as a flagship case study of sexual conflict, within papers from the field and introductory texts alike (e.g. Fricke *et al*., 2009a; Westneat & Fox, 2010; Shuker & Simmons, 2014; Wyatt, 2014; Hosken *et al*., 2019).

Wigby & Chapman's (2005) paper was the final piece of the costs‐of‐mating puzzle that had been building since the late 1980s. Several follow‐up studies provided additional support. First, demonstrations of the fitness benefits males accrue from SP transfer reinforced the sexual asymmetry of SP's fitness effects (Fricke *et al*., 2009b; Wigby *et al*., 2009). A second and perhaps more important discovery in this respect was that the *SPR* gene pre‐dates *SP*. Although *SP* appears restricted to a subset of drosophilids (*D. virilis* is the most distant *D. melanogaster* relative in which an *SP* ortholog has been detected), *SPR* is present in species across the Lophotrochozoa and Ecdysozoa (Kim *et al*., 2010). *SPR*'s presence in the absence of *SP* was explained by the discovery that SPR also interacts with a widely conserved class of ligands called myoinhibitory peptides (MIPs) that have wide‐ranging effects on physiology and behaviour across taxa (Kim *et al*., 2010; Poels *et al*., 2010; Lange *et al*., 2012). For example, in marine annelids, interactions between MIPs and SPR orthologues regulate the settlement of free‐swimming larvae (Conzelmann *et al*., 2013). MIPs are not transferred in the *Drosophila* male ejaculate, nor do they stimulate post‐mating responses in females (Kim *et al*., 2010; Poels *et al*., 2010). MIPs therefore appear to represent the ancestral ligands of SPR. For students of sexual conflict, the significance of the MIP–SPR–SP story was clear. It represented an apparent case of sensory exploitation, not at the macro level of acoustic calls or flashy plumages, but at the molecular level, with SP having evolved to exploit SPR's ancestral function (Poels *et al*., 2010; Coast & Schooley, 2011; Brockhurst *et al*., 2014; Tsuda *et al*., 2015; Tsuda & Aigaki, 2016; Schenkel *et al*., 2018).

**Fig. 2:**
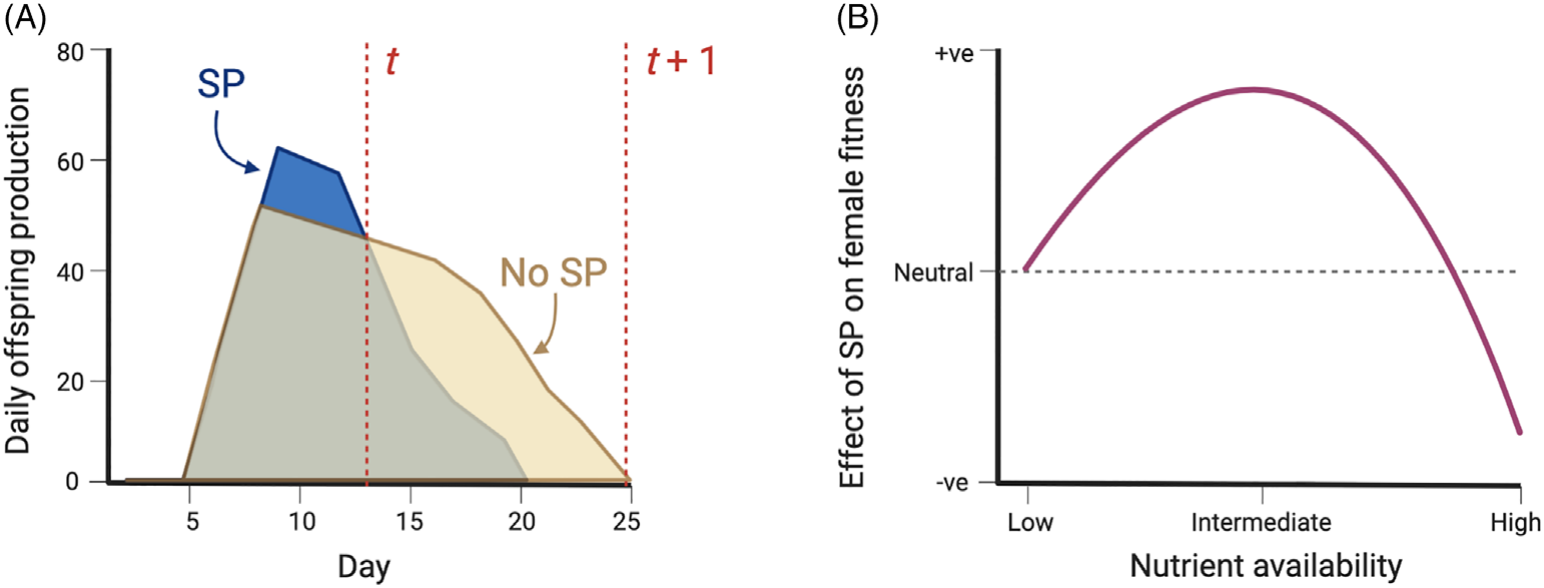
(A) Hypothetical fitness curves that illustrate how the fitness benefits of short‐term support of female reproduction by sex peptide (SP) could be offset by reduced long‐term gains (e.g. due to accelerated reproductive ageing or early death). A female that receives SP (blue) initially shows a greater rate of offspring production compared to a female that does not (gold). However, the female that does not receive SP continues to produce offspring over a longer period, perhaps due to a longer lifespan. The conclusion we reach about whether SP increases or decreases female fitness will depend on whether we measure female offspring production up to, or the female dies at, point *t* or *t* + 1. (B) The weight of evidence is consistent with a model by which the sign and magnitude of female fitness outcomes from receipt of SP depend on the quality of a female's diet. A positive effect is experienced on intermediate diets with neutral effects under limited nutrient availability and negative effects at excess nutrient availability (Wigby & Chapman, 2005; Rogina, 2009; Fricke *et al*., 2010). The effects of SP are presented relative to females that do not receive SP.

The conflict paradigm proposes that females would have higher fitness if they never receive SP. But it does not preclude positive effects of SP on individual fitness components (e.g. Sirot *et al*., 2015; White *et al*., 2021). For example, females might benefit from immune stimulation, increased early‐life fecundity or changes to gut activity that support reproduction induced by SP. But such benefits might be offset by the costly loss of remating opportunities from reduced sexual receptivity or reduced lifespan from SP (Fig. 2A). Ultimately, it is the net effects of SP on female fitness that will determine how selection acts on female responses to it.

## III COEVOLUTIONARY MODELS OF THE ORIGIN AND MAINTENANCE OF SP

Any of several non‐exclusive models might explain the evolution of SP's effects on females. Firstly, SP might have arisen for an ancestral function (such as mediating the transfer of other SFPs; see Sections IV.3 and V.2), and females might subsequently have adopted SP as a cue of ejaculate receipt (Manning, 1967; Chapman, 2001; Brockhurst *et al*., 2014), evolving sensory apparatus that feeds the detection of SP into the induction of reproductive processes. In the language of animal signalling, ‘cues’ provide information, but they have not been shaped by natural selection to do so; instead, they evolve for other functions (Maynard‐Smith & Harper, 2003; Laidre & Johnstone, 2013). Secondly, when SP arose to serve its ancestral function, its expression might have been condition dependent such that the quantity of SP in a male's ejaculate provided a cue of his quality (e.g. if high‐quality males produced bigger ejaculates; e.g. Macartney *et al*., 2019). In this case, females might have evolved sensory apparatus and neural circuitry not only to switch on reproductive processes upon detecting SP, but also to use the information about male quality that SP provides to make reproductive decisions. This would provide a physiological mechanism for tailoring reproductive investment and remating decisions to partner quality (Jennions & Petrie, 2000; Kokko & Mappes, 2005). A third possibility is that SP evolved through sensory exploitation. SP might have evolved to exploit pre‐existing MIP–SPR interactions and manipulate female reproductive processes to the male's advantage (Brockhurst *et al*., 2014; Tsuda *et al*., 2015; Tsuda & Aigaki, 2016; Schenkel *et al*., 2018). Females might then evolve resistance to manipulation (e.g. through increased specificity in MIP–SPR binding), or might be hindered from evolving resistance by strong selection on ancestral SPR functions unrelated to SP.

From these starting points, several evolutionary trajectories are possible. First, selection might act to the mutual benefit of males and females. Whenever receiver responses benefit both signaller and receiver, selection should favour features that promote the efficacy with which the cue or signal elicits the favourable response [‘efficacy selection’ (Guilford & Dawkins, 1991; Scott‐Phillips *et al*., 2012)]. In the case of a cue, the shift to selection acting on the trait for its communicative, rather than ancestral, function marks the transition from a cue to a signal, a process known as ritualisation (Maynard‐Smith & Harper, 2003). By this process, SP might have evolved to be detected more readily by females, perhaps through an increased quantity transferred or an increased binding affinity to SPR or to sperm. Likewise, females might have evolved to detect SP better; for example, by increasing the expression of *SPR* within reproductive tissue, or changing the regulation of as‐yet‐unidentified enzymes that cleave SP from the surface of sperm. Ritualisation may have also instigated the evolution of condition‐dependent expression of SP, if it was not ancestrally condition dependent. The changes that make SP more detectable (e.g. an increased quantity transferred) might elevate costs for males, such that only high‐quality males can transfer sufficiently stimulating quantities of SP. As described above, females might then gain by tailoring reproductive investment and decisions in relation to information on male quality from SP.

Second, selection might act antagonistically. Once females evolve tailored, dose‐dependent responses to SP, there is scope for males to evolve exploitation of the female response by supplying more SP and thereby triggering a female response beyond the female optimum (e.g. see Eberhard, 1996; Arnqvist & Nilsson, 2000; Johnstone & Keller, 2000; Arnqvist & Rowe, 2005; Rowe *et al*., 2005; Arnqvist, 2006; Brockhurst *et al*., 2014). Several coevolutionary trajectories are then possible (Rowe *et al*., 2005). First, females might increase the threshold dose of SP required to trigger a response, generating selection on males to increase SP transfer to surpass that threshold and resulting in a sexually antagonistic arms race. If males evolve increased SP transfer, then the costs of SP production might become so high that only high‐quality males can afford the levels required to manipulate female responses, such that sexually antagonistic coevolution gives rise to a condition‐dependent signal. Second, instead of an increased response threshold, females might evolve decreased sensitivity to SP, moving their response to SP closer to its optimum but limiting selection on males to transfer even more SP in response. With either case – an increased threshold or decreased sensitivity – female resistance might lead to female dependence on SP to stimulate their optimal response. In this case, the transfer of SP would serve the interests of both sexes, marking a transition from exploitation to cooperation (as suggested for intra‐pair displays; Servedio, Price & Lande, 2013; Servedio *et al*., 2019). Conversely, selection may act antagonistically within a condition‐dependent signalling system, as males gain short‐lived advantages from misrepresenting their quality and exploiting females before resistance evolves and the honesty of the system is reinforced (Krakauer & Johnstone, 1995). As these possible trajectories illustrate, it is likely that antagonistic and mutually beneficial selection will make joint and fluctuating contributions to the evolution of SP (as with any reproductive trait; Arnqvist & Rowe, 2005).

## IV DOES SP HARM FEMALES?

In Section II, we described the development of the idea that SP is a source of sexual conflict and highlighted the central position of SP in sexual conflict research. In Section III, we zoomed out from the conflict paradigm to outline the broader landscape of theory describing the origin and maintenance of sexually selected traits. In this section, we ask where the evolution of SP sits in that landscape. We question the strength of evidence for the idea that SP causes net fitness costs for females in contemporary populations, on three grounds. First, results from studies of the effects of SP on female fitness are mixed, and generally report neutral or even positive effects. Secondly, whether any reduction in female fitness from SP manifests in wild populations remains untested. Finally, it is unclear whether studies of SP's fitness effects have been confounded by artefacts of genetic manipulation.

### (1) Effects of SP on female lifetime reproductive success

The strongest evidence that receipt of SP harms females (and the evidence on which its star status in the sexual conflict literature is based) comes from a series of laboratory studies in *D. melanogaster*, beginning with the discovery that females experience costs from mating that are independent of offspring production (see Section II.1) and culminating in Wigby & Chapman's (2005) finding that SP alone could induce the cost of mating (see Section II.3).

Although a negative effect of SP on female lifespan has since been replicated in a study that did not measure reproduction (Tower *et al*., 2017), the sexually antagonistic picture of SP is contradicted by other studies that have directly tested its effects on female fitness. In one study, females from two wild‐derived populations (Melbourne and Innisfail) actually benefitted from continuous exposure to SP‐transferring males, producing more offspring throughout life than females that did not receive SP (Wensing & Fricke, 2018). In a third population – the Dahomey strain in which Wigby & Chapman (2005) found costs from SP – SP had only neutral effects on female fitness. These neutral or positive effects on lifetime reproductive success persisted despite Dahomey and Melbourne females suffering reduced lifespan when held with SP‐transferring males; and Innisfail females actually lived longer when held with SP‐transferring males rather than SP null males (Wensing & Fricke, 2018). Two further studies have shown that females benefit from receiving SP and that the magnitude of benefit depends on the nutritional environment. Females that develop on very limited diets show no decrease in lifetime reproduction in response to SP, although they have reduced lifespan; by contrast, females that develop on medium‐ and high‐quality diets experience higher survival and reproductive success from SP. These results hold both when females receive SP through mating (Fricke *et al*., 2010) or through a genetic manipulation that induces constitutive SP expression within female bodies (Rogina, 2009). Hence, none of these studies have replicated the female fitness costs from SP that made it a focal case study for sexual conflict.

What could explain the discrepancy between the female costs from SP reported by Wigby & Chapman (2005) and the benefits reported by later studies? One hypothesis is that nutrition affects female ability to respond to SP (Perry & Rowe, 2015; Chapman, 2018), and that in very limited food conditions, females suffer costs from attempting to maintain a response to SP that would be adaptive in more permissive food conditions. This hypothesis is consistent with the observation of neutral (rather than positive) effects of SP on female fitness under extreme food limitation (as in Rogina, 2009; Fricke *et al*., 2010). But Wigby & Chapman (2005) report costs for females maintained on a high‐protein (live yeast) diet. Likewise, females ate high‐protein diets in earlier experiments that showed that seminal fluid products mediate the cost of mating (Chapman *et al*., 1995). Perhaps, then, SP's effects on females follow a bell‐shaped function: beneficial on intermediate diets and neutral or harmful at the extremes (Fricke *et al*., 2010) (Fig. 2B). Such a function could arise from malnutrition on low‐protein diets, inducing costs from responses to SP that cannot be supported, and toxicity from excess protein on high‐protein diets (Min *et al*., 2007; McCracken *et al*., 2020), where SP‐induced changes to the gut and metabolism (Reiff *et al*., 2015; White *et al*., 2021) might exacerbate the negative effects of a high‐protein diet. Equivalent bell‐shaped functions have been reported for the relationship between adult male nutrition and male reproductive success (Fricke, Bretman & Chapman, 2008). Under this hypothesis, the finding that females of different genetic backgrounds vary in how strongly SP affects their fitness (e.g. Wensing & Fricke, 2018) could be explained by genetic differences in female nutritional sensitivity.

On balance, it seems that the most parsimonious conclusion from this body of research is that females typically experience net gains in lifetime reproductive success from SP exposure. Negative effects from SP on fitness appear limited to one genetic background under extreme nutritional conditions (Fig. 2B), and even then negative effects are inconsistent across studies. If SP plays a role in sexual conflict, then it seems that the harm it imposes on female fitness manifests only under certain limited circumstances.

### (2) Fitness effects of SP in natural populations

As we have discussed, laboratory studies suggest that if SP harms females, it does so under only particular nutritional conditions. But should we expect laboratory levels of harm to manifest even where the conditions are met? After all, costs and benefits measured in the simplified laboratory environment might not reflect those of the field (Cordero & Eberhard, 2003; Eberhard *et al*., 2003; Eberhard, 2009). One reason for concern is the evidence for a much shorter adult lifespan for *D. melanogaster* in natural populations, compared with laboratory conditions. Adult survival in *Drosophila* has been estimated to be as low as 45–85% per day in capture–recapture experiments, suggesting an adult life expectancy of 1.3–6.2 days in field conditions (Rosewell & Shorrocks, 1987; but see Behrman *et al*., 2015). It is possible that most females in natural populations die before the point of divergence in the lifespans of SP‐receiving and non‐SP‐receiving females (Fig. 2A). If so, then females could reap early‐life reproductive benefits from SP without paying later‐life costs from reduced lifespan (Bonduriansky, 2014). Indeed, female lifespans in natural populations appear likely to be truncated well before any lifespan costs from SP arise. Wigby & Chapman (2005) reported a reduction in female lifespan from 24 to 22 days from SP in one replicate, while in another, both SP‐receiving and non‐SP‐receiving females had median lifespans of 24 days. Tower *et al*. (2017) reported that females that mated with *SP*‐null males had median lifespans of 70 and 80 days across two replicates, while females that mated with wild‐type males had median lifespans of between 28 and 49 days across the two replicates.

There are other ecologically relevant factors that laboratory‐based fitness measures might not account for. One factor is the costs and benefits from the reduced female mating rate that SP induces. Less‐frequent mating might be costly because it prevents females from gaining the benefits of polyandry (Arnqvist & Nilsson, 2000), and laboratory studies that do not allow polyandry would fail to measure this benefit. However, it is not clear that female *D. melanogaster* experience benefits from polyandry. A number of laboratory‐based studies have failed to detect either direct or indirect benefits (Pitnick, Spicer & Markow, 1997; Brown *et al*., 2004; Orteiza *et al*., 2005; Priest, Roach & Galloway, 2008; Stewart *et al*., 2008; but see Castrezana *et al*., 2017 for some evidence of indirect benefits of polyandry in this species). Of course, it is likely that these studies have missed benefits and costs from polyandry that occur in a natural setting; for example, if variance in male quality is higher in natural populations than in the laboratory, then so is the scope for females to benefit from polyandry (e.g. by ‘trading up’; Halliday, 1983). Mating in a natural setting is also likely to engender new costs that a reduced remating rate would protect from, such as the risk of predation during copulation (Fairbairn, 1993; Rowe, 1994) and from sexually transmitted infections (STIs) not present in laboratory populations (e.g. Miest & Bloch‐Qazi, 2008).

Another factor is the fitness consequences associated with female immune responses to SP. SP stimulates antimicrobial gene expression in the female reproductive tract, but suppresses resistance to systemic infection (Schwenke, Lazzaro & Wolfner, 2016; Schwenke & Lazzaro, 2017). Prioritising the allocation of resources to localised rather than systemic immune responses might be adaptive in the presence of STIs in the field, but deleterious if they are absent in the laboratory. Similarly, the increased female activity and aggression induced by SP might benefit females in nature, but impose costs in enclosed environments (Bath *et al*., 2017; Malek & Long, 2019). Identifying whether laboratory conditions under‐ or overestimate the costs and benefits of SP receipt is critical to understanding the evolution of SP and the operation of sexual conflict more broadly (Fricke *et al*., 2009a; Perry & Rowe, 2018).

### (3) Can phenotypes associated with SP mutants be definitively attributed to SP?

**Fig. 3:**
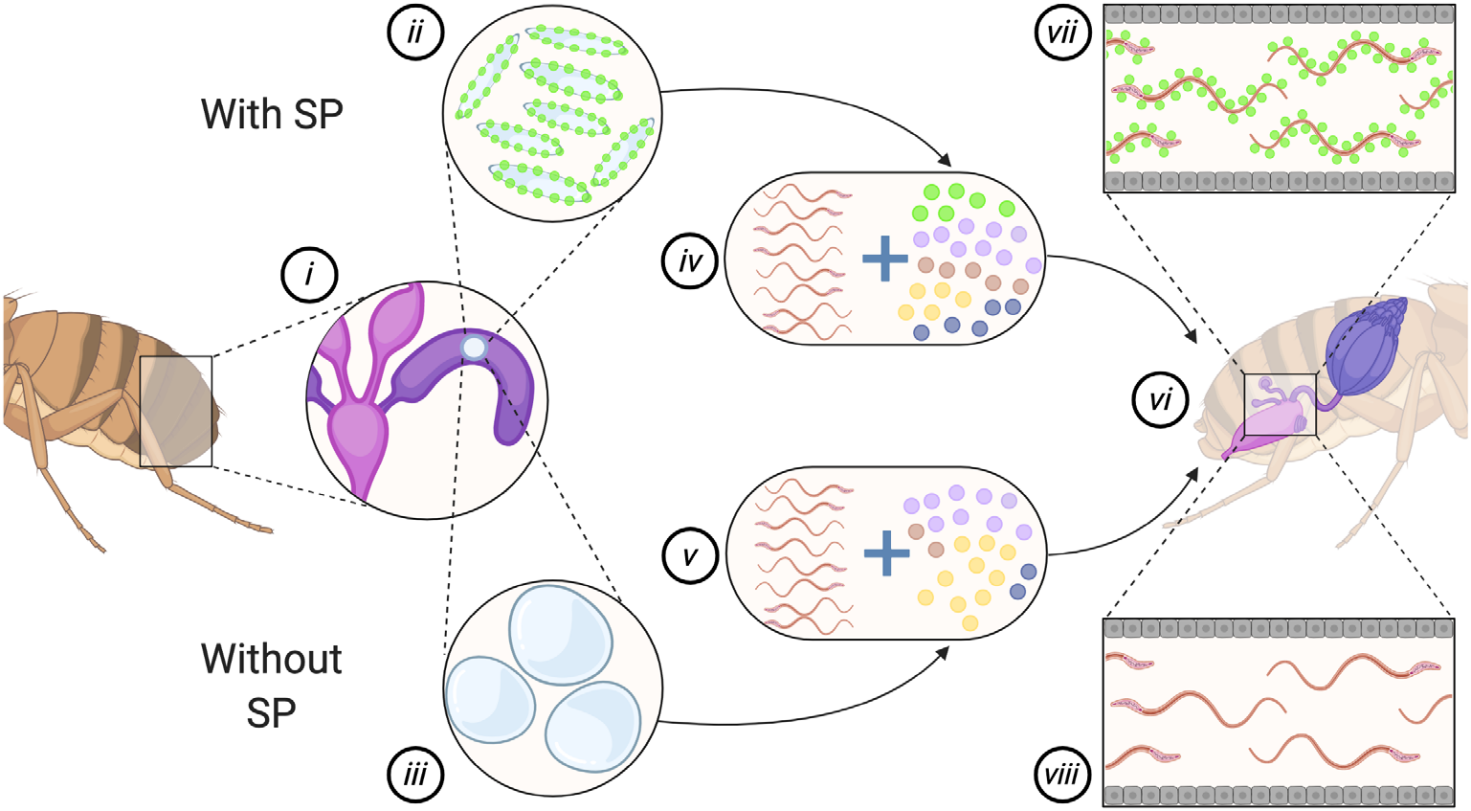
Loss of sex peptide (SP) disrupts the structure of seminal microcarriers and changes seminal fluid composition (Wainwright *et al*., 2021). (*i*) SP is produced in the male accessory glands, a pair of seminal fluid‐producing glands that branch off from the ejaculatory duct at the base of the testes. (*ii*) Within the lumen of the male accessory gland, SP (green) is carried on lipid‐containing microcarriers. Microcarriers are transferred to females during mating and rapidly disassemble within the female reproductive tract. SP is integral to their normal assembly and disassembly. (*iii*) In the absence of SP, microcarriers fail to form correctly and are highly enlarged. (*iv*) In normal matings, males transfer a suite of seminal proteins (coloured circles), including SP, and sperm to females. (*v*) When SP is absent, the dysfunction in microcarriers leads to changes in the composition of the seminal proteome. Some seminal fluid proteins (SFPs) are transferred in greater quantities, others are reduced. (*vi*) Seminal proteins and sperm are transferred to the female. (*vii*) In normal matings, some SP enters directly into the female haemolymph shortly after mating. SP also binds to the surface of sperm, a process facilitated by a network of male‐derived, co‐factor ‘network proteins’. Sperm, along with bound SP, are transported into the storage organs: the seminal receptacle and paired spermathecae. Here, SP is required for the long‐term, multi‐day maintenance of post‐mating responses, as well as the normal release and use of sperm from storage. (*viii*) Although sperm accumulation into storage is not affected by the absence of SP, the subsequent release of sperm is reduced and the duration of post‐mating responses is truncated (Avila *et al.,* 2010).

Can we be confident that the fitness effects attributed to SP are in fact caused by SP? A recent study has revealed an unexpected role for SP within ejaculates: SP is critical to the formation and dissipation of ‘microcarriers’, which are lipid‐based structures that provide storage, delivery, and dispersal machinery for several seminal proteins transferred to females (Wainwright *et al*., 2021) (Fig. 3). This finding brings into question the extent to which the effects observed using SP knockouts and knockdowns can be attributed to SP alone, rather than the disrupted transfer of a broader set of seminal proteins. Indeed, ectopic expression studies have detected toxic effects on females from several SFPs, including CG10433, CG8137, and Acp62F (Lung *et al*., 2002; Mueller, Page & Wolfner, 2007). If the transfer of these proteins is disrupted by the loss of SP, then it might be their reduced transfer or absence that causes reduced reproduction or lifespan (where detected) for females that mate with wild‐type males compared with females that mate with SP‐knockdown and ‐knockout males.

Given SP's role in microcarrier formation, the most direct way of assessing its effects on females is through the ectopic expression of SP in females or injection of SP into females. In studies using these methods, SP stimulates juvenile hormone synthesis (Moshitzky *et al*., 1996), feeding (Carvalho *et al*., 2006), immune activity (Peng, Zipperlen & Kubli, 2005b), improvements in memory (Scheunemann *et al*., 2019), ovulation, egg‐laying and reduced sexual receptivity (Chen *et al*., 1988; Aigaki *et al*., 1991; Schmidt *et al*., 1993a; Nakayama, Kaiser & Aigaki, 1997; Soller, Bownes & Kubli, 1997; Haussmann *et al*., 2013; Tsuda *et al*., 2015; Wensing & Fricke, 2018). Hence, reassuringly, studies using these approaches recapitulate many of the phenotypic effects observed in knockdown (Chapman *et al*., 2003b) and knockout (Liu & Kubli, 2003) studies. However, how well do these more targeted delivery methods recapitulate the costs attributed to SP in knockdown (Wigby & Chapman, 2005) and knockout (Tower *et al*., 2017) mating experiments? In the only study using ectopic expression of SP to measure fitness costs, SP increased lifetime egg production across all but a calorie‐restricted diet (Rogina, 2009). In other studies measuring SP's impact on adult female lifespan, mixed effects have been reported. In *otu* ^*6*^ homozygotes, which are unable to produce mature eggs, ectopic SP expression causes a marked reduction in female lifespan (Ueyama & Fuyama, 2003). But in the fertile heterozygotes, this trend is reversed: ectopic expression of SP leads to a significant extension of lifespan. Thus, the negative effects of ectopic expression appear to be restricted to females defective in oogenesis. However, ectopic studies suffer from their own limitations, expressing SP under the control of near‐ubiquitously expressed promoters or at high levels (Sirot *et al*., 2015). Variation in the identity of these promoters may account for differences among studies. The demonstration of high larval mortality from ectopic SP expression by Mueller *et al*. (2007) used a ubiquitous *tubulin*‐GAL4 driver, while the diet‐dependent (Rogina, 2009) and genotype‐specific (Ueyama & Fuyama, 2003) costs and benefits of SP were detected using a transgenic construct under the control of the yolk protein enhancer, which should restrict SP expression to the fat body, where it can be secreted into the haemolymph (Aigaki *et al*., 1991). Thus, although SP may have toxic potential when highly and broadly expressed, the sign of these effects appears reversed under more restricted expression.

Relative to ectopic expression, the injection of SP may be better able to mimic the level females receive in mating. Here, however, the injected material is introduced directly into the female haemolymph, which presumably would not normally receive so large a direct dose of SP (Peng *et al*., 2005b). An alternative to these approaches is to evaluate benefits and costs of SP in genetically modified females that do not express the SP receptor (*SPR*), compared with wild‐type females (e.g. Perry *et al*., 2016; Morimoto *et al*., 2019). However, the *SPR* deletion removes several flanking genes, and *SPR* is broadly expressed in the nervous system and interacts with ligands other than SP (see Section II.3), making it difficult to attribute differences to the effects of SP alone. Knockdown of *SPR* in SP‐sensing neurons or their inactivation *via* the GAL4, split‐GAL4, or LexA expression systems has been used to study the function of these neurons (Häsemeyer *et al*., 2009; Rezával *et al*., 2012) and would provide a more targeted approach for studying the fitness consequences of responding to SP.

Overall, each approach – SP knockouts and knockdowns, ectopic expression, injection, and *SPR*‐null females – has limitations that confound assessments of the fitness effects of SP receipt for females. Studies that combine complementary approaches within the same protocol will be most valuable, but for the reasons outlined above, all of these approaches are likely to lead to the misestimation of SP's costs for females. It is therefore useful to consider other ways to evaluate SP's benefit and harm to females, such as the evolutionary genetics and mechanistic basis of SP‐induced phenotypes.

## V THE EVOLUTION OF SP FUNCTION

In the previous section, we argued that the widespread view of SP as a sexually antagonistic molecule fails to fully represent the results of studies of SP's effects on females. In this section, we argue that this view is also inconsistent with what we know of the genes, proteins, and neural circuitry underlying SP's induction of female post‐mating responses.

### (1) Did sensory exploitation drive the evolutionary origin of SP?

The ‘sensory exploitation’ model (Sections II.3 and III) for SP's evolution accounts for the observations that (*i*) the *SPR* gene long pre‐dates *SP*, and (*ii*) *SPR* has another class of ancient, conserved ligands (MIPs) to which it binds (Kim *et al*., 2010; Poels *et al*., 2010). The model is further bolstered by studies showing that *D. melanogaster* SP can stimulate post‐mating changes in a distant relative (the moth *Helicoverpa armigera*; Fan *et al*., 1999), and, in *in vitro* assays, bind to the SPR orthologs of other distant relatives that similarly lack an SP gene (the mosquito *Aedes aegypti* and the silkworm *Bombyx mori*; Yapici *et al*., 2008). These same *in vitro* assays revealed that the binding affinity between *D. melanogaster* SP and its own SPR ortholog was considerably higher than to those of other species, indicating evolutionary change in SPR sequence that rendered it more sensitive to SP (Yapici *et al*., 2008). Collectively, this suggests that SPR has a long‐standing and elevated sensitivity to SP, which – according to the sensory exploitation model – SP evolved to exploit. This model assumes that females are unable to lose or modify SPR to ignore SP without inviting a greater fitness cost from disrupting the ancestral SPR*–*MIP interactions (a ‘sensory trap’; West‐Eberhard, 1979). Consistent with this model, disrupting the action of SPR does appear costly to females, at least in contemporary *D. melanogaster* populations. In a study in which *SPR* was genetically modified to be linked to blindness in males – and therefore associated with the reduced mating success of blind males – *SPR* persisted stably over many generations in laboratory populations (Dean *et al*., 2012). It could only have been maintained, despite costs incurred by blind males, through benefits conferred by *SPR* on females.

On these observations, the case for SP's evolution through sensory exploitation seems strong. However, a closer look at SPR's expression within the reproductive tract, and its evolution, reveals important inconsistencies with sensory exploitation.

#### (a) SPR expression within the female reproductive tract evolved after SP, not before

**Fig. 4:**
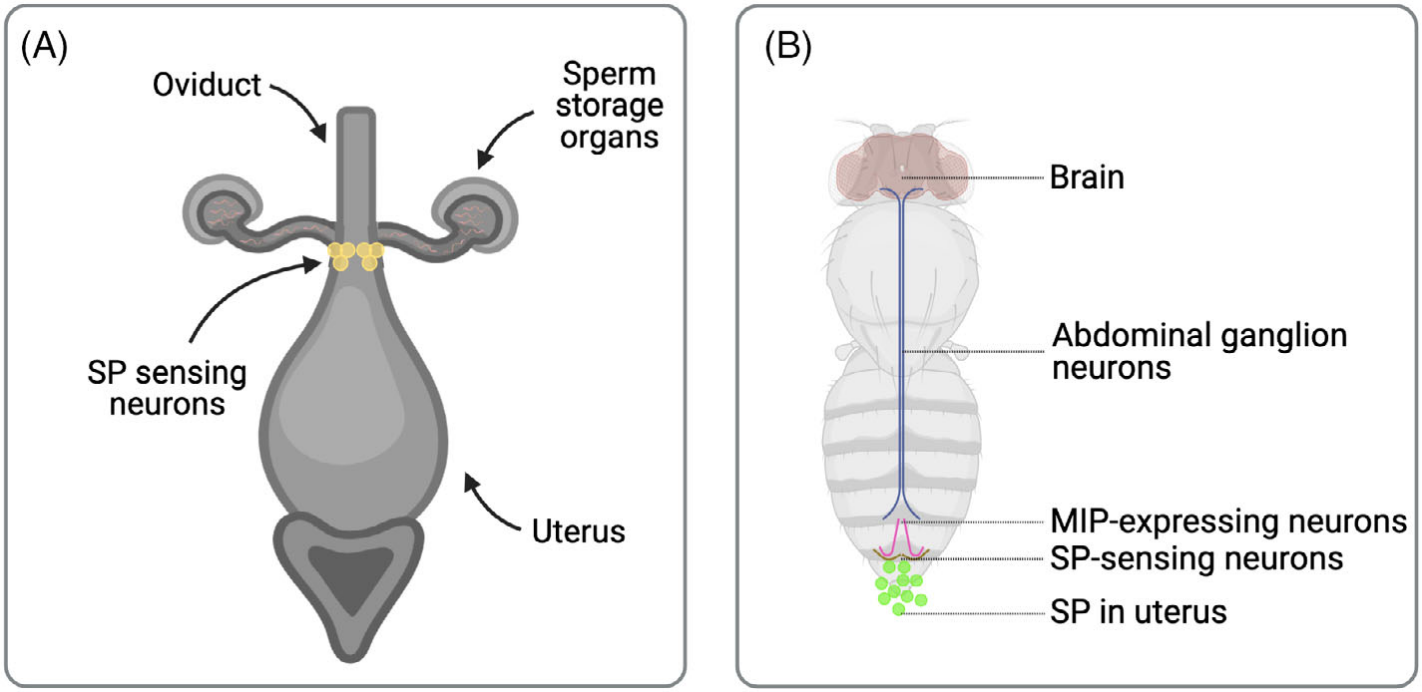
(A) The sex peptide (SP)‐sensing apparatus within the female reproductive tract consists of two bilateral clusters of three sex peptide receptor (SPR)‐expressing neurons (Häsemeyer *et al*., 2009; Yang *et al*., 2009; Rezával *et al*., 2012). These neurons are necessary and sufficient for the reduction in receptivity and stimulation of egg‐laying induced by SP and co‐express *doublesex*, *fruitless*, and *pickpocket*. Figure redrawn and modified from Rezával *et al*. (2012). (B) A schematic of the neural circuitry underlying the female post‐mating response, modified from Jang *et al*. (2017). SP enters the uterus where it is detected by SP‐sensing neurons (see A). Myoinhibitory peptide (MIP)‐expressing interneurons relay the detection of SP to SP abdominal ganglion neurons, which in turn extend to the brain.

The first inconsistency relates to SPR's spatial expression pattern within females. The core elements of the female post‐mating response (stimulated oviposition and reduced receptivity) are induced by a set of two bilateral clusters of three *SPR*‐expressing, SP‐sensing neurons in the uterus near the sperm storage organ ducts (Häsemeyer *et al*., 2009; Yang *et al*., 2009; Rezával *et al*., 2012) (Fig. 4A). MIP‐expressing neurons then relay SP detection from these neurons in the reproductive tract to neurons that project into the brain (Jang, Chae & Kim, 2017) (Fig. 4B). While it is easy to imagine that losing *SPR* from the central nervous system, where it is expressed in both males and females (Yapici *et al*., 2008), might cause significant, costly disruption to ancestral MIP–SPR interactions, losing expression in this small cluster of neurons near the uterus might cause negligible disruption. If responding to SP reduces female fitness, or has done so in the past, then this cluster of neurons is unfortunately positioned from the female's perspective, sitting where they are likely to encounter the highest concentrations of SP, both after mating and for the duration of sperm storage.

A more complete sensory exploitation model predicts not only that *SPR* pre‐dates *SP*, but specifically that *SPR* expression in reproductive tract neurons pre‐dates *SP*. However, a recent study contradicts this prediction. Instead, it suggests that *SPR* expression in the reproductive tract is a derived trait, and that *SP* evolved before robust expression of *SPR* in the female reproductive tract (Tsuda *et al*., 2015). *SPR* is expressed in non‐reproductive tissues of both sexes across all *Drosophila* examined to date, but robust expression of *SPR* in the female reproductive tract, and the ability of SP to bind to SPR in this region, is an innovation confined to the *melanogaster* species group (Tsuda *et al*., 2015). This is despite the presence of *SP* orthologues outside the group (Tsuda & Aigaki, 2016; McGeary & Findlay, 2020). Crucially, species with *SP* orthologues but without *SPR* expression in the female reproductive tract (including *D. persimilis*, *D. pseudoobscura*, and *D. willistoni*) do not show the key post‐mating response of reduced receptivity when injected with SP (Tsuda *et al*., 2015). These observations suggest that the extension of *SPR* expression to the female reproductive tract evolved only after SP orthologues evolved, presumably through female benefit from detecting and responding to SP. Hence, SP does not appear to have evolved to exploit a constitutive SPR–MIP system, as the sensory exploitation model proposes; rather, the crucial innovation appears to be the evolution of expression of *SPR* in just a few sensory neurons in the female reproductive tract. Moreover, it suggests that the regulation of *SPR* expression is sufficiently evolutionarily labile that, if responding to SP is deleterious to females, females could evolve the loss of *SPR* expression in the SP‐sensing neurons in the reproductive tract without disrupting important MIP–SPR interactions in other tissues.

#### (b) SPR's sensitivity to SP can evolve without disrupting SPR–MIP interactions

A sensory trap model predicts that females have only limited scope to escape SP's effects because changes in SPR that limit SP‐binding would incur costs from disrupted MIP–SPR interactions. However, it appears that at least some SPR residues can evolve to confer differential sensitivity to SP and MIP ligands. The SPR sequence of *D. melanogaster* and its close relatives includes a QRY motif that replaces the well‐conserved DRY sequence found in many invertebrate G‐protein‐coupled receptors (Poels *et al*., 2010). Interestingly, *in vitro* studies have shown that reintroducing the DRY motif into *D. melanogaster* SPR renders it less responsive to SP, while the response to MIPs is much less affected (Poels *et al*., 2010). Similarly, replacing the proline residue at position 238 in *D. melanogaster* SPR with the corresponding leucine residue from the SPR of the sea slug *Aplysia californica* reduces sensitivity to SP 2.7‐fold without reducing sensitivity to MIPs (Lee *et al*., 2020). Thus, the consequences of change at some SPR residues for SP and MIP interactions can be decoupled, providing a route for SPR to evolve changes in sensitivity to its different ligands with some independence and suggesting that SPR might escape a sensory trap.

### (2) Ancestral functions for SP

If, as we have argued above, SP did not evolve to exploit an existing sensory predisposition in the female reproductive tract, then what did it evolve to do? A possible ancestral function that is independent of SPR is SP's role in the formation and activity of microcarriers, a storage, delivery, and dispersal machinery for seminal products (see Section III; Wainwright *et al*., 2021). This ancestral role is supported by the observation that the presence of microcarriers in other *Drosophila* species appears to co‐vary with the *SP* gene; for example, *D. mojavensis*, which lacks *SP*, does not produce microcarriers, while *D. pseudoobscura* and *D. persimilis*, which have SP, do (Wainwright *et al*., 2021). Furthermore, although the latter species produce SP, they lack clear female post‐mating responses and do not express *SPR* in the female reproductive tract (Tsuda *et al*., 2015). Thus, the ancestral function of SP may be in structuring microcarriers rather than in mediating post‐mating responses.

A further possibility is that SP initially evolved to target receptors (SPR or others) expressed elsewhere in the female. Consistent with this hypothesis, some female post‐mating responses to SP appear to be independent of SPR (Haussmann *et al*., 2013). For example, the increase in female aggression after mating, which is partly mediated by SP, is as pronounced in *SPR‐*null females as in controls (Bath *et al*., 2017). In *D. melanogaster*, SP passes into and is processed in the female haemolymph, where it could reach neuronal targets (Moshitzky *et al*., 1996; Pilpel *et al*., 2008; Ravi Ram & Wolfner, 2009; Haussmann *et al*., 2013). Thus, SP might affect females even in species lacking *SPR* expression in the reproductive tract. To resolve the order of evolutionary events, it will be important to test whether female responses to SP that occur in the absence of SPR, such as the post‐mating stimulation of aggression (Bath *et al*., 2017), are also present in species lacking robust expression of *SPR* in the female reproductive tract.

### (3) SP as a cue or signal of female sperm receipt and storage

In Section III, we described an evolutionary route by which a cue could become ritualised into a signal through selection acting on signallers or receivers to increase a cue's detectability (Maynard‐Smith & Harper, 2003). The order of evolutionary transitions in the SP–SPR system observed within the *Drosophila* phylogeny is consistent with this process. Following the evolution of SP transfer, the subsequent evolution of *SPR* expression in the female reproductive tract may have enhanced female ability to detect SP and to use its detection to switch to a reproductive state more quickly and accurately. This puts mechanistic meat on the bones of the long‐standing idea that females could use SP (and SFPs in general) as a cue of successful mating (Manning, 1967; Eberhard, 1996; Brockhurst *et al*., 2014).

In the *D. melanogaster* female reproductive tract, SP binds to the surface of sperm, enters the female storage organs, and is slowly released, prolonging female post‐mating responses (Peng *et al*., 2005a; Ravi Ram & Wolfner, 2007, 2009; Singh *et al*., 2018). The ability of SP to bind to sperm provides a powerful mechanism for coupling the maintenance of post‐mating responses to the presence of stored sperm. In this sense, the SFP cofactors that facilitate SP–sperm binding act as ‘amplifiers’ of SP, features that are secondary to the signal itself that serve to enhance its detection as some plumage and display features do for ornaments in birds (Hasson, 1989, 1991). It is possible that even SFPs outside of the SP network play amplifying roles, perhaps reconfiguring the reproductive tract environment to maximise the detection of SP.

When SP's sperm‐binding capability evolved is unclear, but both antagonistic and cooperative routes are possible. For females, the binding of SP to sperm, and therefore its long‐term entry into female storage organs, could entail costs if it prolongs post‐mating responses induced by SP that benefit females only in the short term. Conversely, SP–sperm binding could benefit females by providing a mechanism for maintaining a reproductive state only as long as sperm are stored. If SP is differentially bound and released by live and dead sperm, then the detection of SP might further provide specific information on the presence of live sperm in storage. Sensitivity to the quantity of SP in storage might also allow females to ‘count’ sperm as they enter or exit storage, and thereby assess the utility of remating to acquire more sperm (Gromko & Markow, 1993; Kohlmeier *et al*., 2021; Perry & Hopkins, 2021). These ideas are consistent with the specific localisation of SP‐sensing neurons, near where the spermathecae meet the uterus (Rezával *et al*., 2012) (Fig. 4A). If females use SP to ‘count’ sperm in the reproductive tract and tailor their post‐mating responses accordingly, then males could gain from transferring more SP to induce the female to overestimate the sperm transfer and to remain unreceptive to remating for longer. In turn, females should gain from detecting any dishonest representation of sperm numbers. Females would gain most from relying on information about sperm number that is least susceptible to male dishonesty, such as an SFP that is bound to sperm. It would therefore be interesting to learn whether SP's binding to sperm evolved for an ancestral function before females evolved the expression of *SPR* within the reproductive tract, consistent with females evolving to use SP as a cue or signal of sperm receipt.

If SP is used to signal the storage of sperm, then we should expect SP's effects on female reproduction to co‐vary with the duration of sperm storage. Some species that transfer giant sperm remate at an exceptionally high rate (e.g. four times in a morning in *D. hydei*; Markow, 1985) that is likely at least partly due to females receiving very few sperm per mating (Lüpold *et al*., 2016). Species like these may have little need for sensing and responding to SP due to the brief period of sperm storage. Consistent with this idea, SP has not been detected in *D. repleta*, the closest‐studied relative of *D. hydei* (McGeary & Findlay, 2020) or in our own search of the reference genome for *D. hydei* (following the search strategy employed by McGeary & Findlay, 2020). It would be fruitful to test for associations across the *Drosophila* phylogeny between the presence of sperm‐binding SP, female expression of *SPR* in the reproductive tract, and the duration of sperm storage. This approach might also help to resolve the evolutionary order of events in *repleta*‐group species with rapid remating: do they remate frequently because they lack SP, or can they dispense with SP because they remate frequently?

The ability of SP to bind to sperm raises a critical question: who controls the release of SP from the surface of sperm? The extension of post‐mating responses requires not just the binding of SP to sperm and its entry into storage, but also its subsequent release. Transgenic *D. melanogaster* males missing the trypsin cleavage site in the SP sequence bind permanently to sperm and only short‐term post‐mating responses are elicited (Peng *et al*., 2005a). Under a sexual conflict perspective, this represents a crucial battleground: whoever produces the as‐yet unidentified trypsin‐like enzyme that cleaves the C‐terminus of SP from sperm controls whether post‐mating responses last for 1 day or over 10. It is possible that males transfer this enzyme and it remains stored within the female reproductive tract along with sperm and SP. But a more parsimonious scenario is that it is produced by the female. The question then is: do males exploit an ancestral, female‐derived trypsin with pleiotropic functions in the reproductive tract, or do females secrete the trypsin because of the benefits associated with freeing SP? Based on the arguments raised throughout this review, the latter appears most likely.

### (4) SP as a signal of male quality

Females might use SP as a cue or signal of sperm receipt or storage, but another, non‐mutually exclusive function is possible: SP may signal male quality or condition. SP might have been ancestrally condition dependent, before the evolution of any signalling function (Biernaskie, Perry & Grafen, 2018), or condition dependence could have evolved secondarily, as a result of exaggeration through either sexually antagonistic coevolution or a mutually beneficial process of ritualisation (Section III). In either case, females may gain by tailoring their reproductive effort to quantitative or qualitative variation in the SP they receive, if there are indirect benefits to be gained from producing offspring sired by high‐condition males (Long, Agrawal & Rowe, 2012). Whether females experience net fitness gains from their response will depend in part on the route through which condition dependence evolved. If condition dependence has arisen from exaggeration through sexually antagonistic coevolution, then theory suggests that any indirect benefits from responding to male condition are unlikely to outweigh direct costs of SP receipt (Kirkpatrick & Barton, 1997; Cameron *et al*., 2003).

For SP to function as a condition‐dependent signal, the quality or quantity transferred must co‐vary with male quality or condition, and females must tailor their reproductive decision‐making in response to variation in SP (i.e. a dose‐dependent response) and benefit from doing so. If SP functions as a signal of sperm storage alone, then females must also benefit from responding to SP. However, in this case the quantity of SP transferred may or may not reflect male quality, and females may either show a threshold response to SP, initiating post‐mating responses once a minimum quantity is detected, or a dose‐dependent response. A dose‐dependent response to SP as a sperm storage signal could occur if SP transfer is tightly linked to sperm number, and females benefit from tailoring the duration or total stimulation of post‐mating responses to the number of sperm received (see Section V.3). Because both the hypotheses of SP as a signal of sperm number or of male quality are consistent with dose‐dependent female responses, they may be difficult to distinguish, and if sperm number itself indicates male quality, they may be equivalent. Overall, if SP transfer is condition dependent, female responses to SP are dose dependent, and females benefit from their responses, then the hypotheses cannot be distinguished, whereas if SP transfer is not condition dependent or female responses to it are not dose dependent, then the hypothesis of SP as a signal of male quality can be dismissed.

#### (a) Is SP transfer condition dependent?

We focus on quantitative variation in SP transfer because it has been studied in more detail than qualitative variation, the scope for which is limited by SP's small size. However, qualitative variation in SP is possible in the form of post‐translation modification (i.e. hydroxylation) of hydroxyproline and an isoleucine residue (Chen *et al*., 1988; Sturm *et al*., 2020). In fact, *D. melanogaster* populations vary in SP hydroxylation (Sturm *et al*., 2020), although the functional consequences are unclear. Post‐translational modification is one mechanism by which SP's structure might reflect male condition (Sturm *et al*., 2020), and hence it is conceivable that females could gain information about male quality from SP's structure. This interesting possibility deserves future tests, particularly because female response to variation in SP quality is a unique prediction of the male quality signalling hypothesis.

There is some evidence that SP production and transfer is condition dependent. Large, high‐condition males have more SP stored in their accessory glands and tend to transfer more to females during mating, relative to small, low‐condition males (Wigby *et al*., 2016). The amount of SP transferred by large males represents a lower proportion of their SP reserves, compared with small males (Wigby *et al*., 2016). Furthermore, males reared on a diet of intermediate quality induce stronger reductions in female receptivity, relative to males reared on a low‐quality diet, consistent with increased transfer of SP by higher‐quality males (although males reared on a high‐quality diet induce weaker post‐mating responses, suggesting that quality dependence might not be monotonic; Fricke *et al*., 2008).

But is the covariance between male condition and quantitative variation in SP production and transfer any greater than for other SFPs, *i.e*. is it SP that is condition dependent or the ejaculate? Wigby *et al*. (2016) showed that SP transfer is more strongly linked to male size (a likely correlate of condition) than the SFP ovulin, but in general there is scant evidence on the condition dependence of SP relative to other SFPs. Moreover, whether SP *should* stand out in the strength of its condition dependence is unclear. The long‐term storage of SP requires SP's co‐factor network SFPs, which facilitate SP's binding to sperm (Ravi Ram & Wolfner, 2007, 2009; Findlay *et al*., 2014; Singh *et al*., 2018), such that perhaps the SP network as a whole should show stronger condition dependence relative to other SFPs. This interdependence between seminal components presents a challenge for the identification of condition‐dependent ejaculate signals.

#### (b) Do females tailor reproductive effort to SP quantity?

Females might tailor their reproductive decisions to SP quantity at any of several stages. Immediately after mating, females might alter their processing of an ejaculate to bias sperm storage towards favoured males. However, there is no evidence that SP influences ejaculate processing. *Drosophila melanogaster* females can eject sperm after mating (Lüpold *et al*., 2013; Lee *et al*., 2015; Hopkins *et al*., 2019a), but there is no evidence that SP influences this process: females mated to *SP*‐null males store equivalent sperm quantities to females mating with SP‐transferring males (Avila *et al*., 2010).

After mating, females might adjust their propensity to remate in response to SP. SP decreases female receptivity to remating, a response often interpreted as male manipulation of female remating (e.g. Hollis *et al*., 2019). However, reduced female receptivity might instead come about because females show ‘enhanced selectivity’ after receiving more SP, remating only when they encounter higher‐quality males (Perry *et al*., 2013). Most experimental tests of female remating do not allow the hypotheses of male manipulation *versus* enhanced selectivity to be distinguished: male condition is usually standardised such that females have little incentive to ‘trade up’, and females are often housed in isolation, depriving them of information about population variation in male quality (reviewed in Laturney, van Eijk & Billeter, 2018). Indeed, it is noteworthy that considerable variation in remating interval has been exposed when female *D. melanogaster* are housed with males immediately after mating (Fricke & Chapman, 2017), but whether this variation is driven by variation in SP transfer remains untested. Crucially, a recent study provided support for the ‘enhanced selectivity’ hypothesis, demonstrating that receipt of SP shifts female preferences to males with richer pheromone profiles (Kohlmeier *et al*., 2021). Whether pheromone‐rich males are higher quality or instead manipulating females into remating against their interests remains unclear; testing whether females gain from preferentially mating with high‐pheromone males will be critical to resolve this. Aside from this, there is more direct evidence consistent with dose dependence in how remating decisions respond to SP quantity. Injection studies suggest that female receptivity to remating continues to decrease at higher SP concentrations (Schmidt *et al*., 1993a; Wensing & Fricke, 2018). On the other hand, there is a surprising quadratic relationship between genetic variation in male *SP* expression and female receptivity to remating: males from low and high *SP* expression isolines induce females to be less receptive to remating, compared with males from isolines with intermediate *SP* expression (Smith *et al*., 2009). Both methodologies suffer limitations: the fidelity with which SP injection recapitulates the effects of SP transferred in the ejaculate is unclear (Section IV.3), and SP transcript abundance and protein transfer are not strongly correlated (Smith *et al*., 2012). A full picture of dose dependence in the relationship between SP transfer and female mating decisions awaits more data. A promising approach is to couple measures of female receptivity with sensitive enzyme‐linked immunoassay (ELISA)‐based quantification of transferred and stored SP, as has been undertaken for female fecundity (see below).

Finally, females might tailor reproductive investment by differentially allocating resources to either offspring number or quality in response to quantitative variation in SP receipt. SP is known to stimulate offspring production (Chapman *et al*., 2003b; Liu & Kubli, 2003), and females receive an added fecundity boost if they remate with a second male soon after a first (an effect that is lost at greater inter‐mating intervals) (Smith *et al*., 2017; Nguyen & Moehring, 2018). These data are consistent with a dose‐dependent female response to SP, but double matings are an imperfect proxy for inferring the effects of greater SP receipt. One reason is that only a subset of females will remate soon after a first mating and this subset may be biased in relation to features of the first ejaculate they received. Moreover, it is unclear whether the dose‐dependent response is caused by SP *versus* other seminal products, sperm, the mechanical stimulation of mating, or all three. Indeed, evidence from other studies – SP injection studies and ELISA‐based quantification of SP transfer – suggest a threshold response instead of dose dependency (Schmidt *et al*., 1993a; Smith *et al*., 2012; Wensing & Fricke, 2018). Collectively, these studies leave open the possibility that further stimulation of fecundity by increased SP quantity is only observed if doses are delivered in quick succession (as in successive matings), rather than at once. It would be worthwhile to test this hypothesis by injecting SP doses of varying concentrations and inter‐dose intervals.

In terms of investment in individual offspring, females produce higher‐fitness daughters if they receive seminal fluid (containing SP) from a second male (Priest *et al*., 2008; see also Garcia‐Gonzalez & Dowling, 2015), consistent with dose dependence. The mechanistic basis of this effect is unclear. Although the effect could be influenced by other seminal proteins, one possibility is that the stimulation of yolk deposition by SP (Soller *et al*., 1997) might be sensitive to SP concentration. By this process, females that receive more SP from a single male might produce larger eggs, akin to the larger eggs produced by female birds paired with attractive males (Horváthová, Nakagawa & Uller, 2011). This represents a higher investment because in *D. melanogaster*, larger eggs are associated with increased viability and development rate (Azevedo, French & Partridge, 1997). That *D. melanogaster* females might differentially allocate through offspring provisioning instead of offspring number is consistent with theory: female differential allocation is predicted to be directed towards offspring number when males contribute direct benefits to females – negligible in flies – but towards offspring size when male quality influences offspring fitness (Kindsvater & Alonzo, 2014), for which there is some evidence in *D. melanogaster* (Rundle, Ödeen & Mooers, 2007). A similar yolk deposition mechanism may explain the finding in another fly species (*Telostylinus angusticollis*) that a female's previous mate can influence the phenotype of offspring sired by subsequent mates (Crean, Kopps & Bonduriansky, 2014; Crean, Adler & Bonduriansky, 2016), although it is likely that this process operates independently of SP, given that SP orthologues appear to be restricted to *Drosophila*.

The possibility that females invest more in offspring provisioning raises an important point for studies of harm to females from SP. If females invest in offspring quality in response to receiving more SP, then the extra energy that this requires might mean that females produce fewer eggs (of better quality) or suffer reduced lifespan. This depression in female reproduction or lifespan would make SP look like a harmful trait, as in Wigby & Chapman (2005) (see also Ueyama & Fuyama, 2003; Tower *et al*., 2017) and in studies of the costs of mating that came before (Fowler & Partridge, 1989; Chapman *et al*., 1993, 1995). Hence, unbiased estimates of fitness effects from SP should extend to the F2 generation, at a minimum.

In sum, although the rate of offspring production appears insensitive to quantitative variation in SP, there is good evidence that female willingness to remate is dose dependent, and some evidence that the per‐offspring rate of provisioning is dose dependent.

#### (c) Practical issues for a seminal fluid signal of male condition

The idea that SP acts as a signal of male condition raises questions about how honesty is maintained, female information processing, and the interpretation of previous experimental evolution studies.

##### (i) Erosion of the link between SP quantity and male condition

Several forces might act to erode positive covariance between male quality and SP transfer. First, the amount of SP a female receives might be a function not only of male quality but also of his exposure to competition. *Drosophila melanogaster* males strategically alter the quantity of sperm and SFPs produced and transferred in response to cues of sperm competition (Bretman, Fricke & Chapman, 2009; Wigby *et al*., 2009; Fedorka, Winterhalter & Ware, 2011; Sirot, Wolfner & Wigby, 2011; Garbaczewska, Billeter & Levine, 2013; Hopkins *et al*., 2019b). These changes do not appear to be uniform across ejaculate components, as expected if ejaculate size was responding to sociosexual cues. Instead, sperm and the seminal proteome appear to show differential sensitivity to levels of competition, and different subsets of SFPs appear to be more responsive than others (Hopkins *et al*., 2019b). Although a quantitative proteomics study failed to find a significant effect of prior exposure to competition on the quantity of SP transferred (Hopkins *et al*., 2019b), a more targeted ELISA‐based study found that males exposed to competitors at the time of mating transferred more SP (Wigby *et al*., 2009). If SP transfer is sociosexually responsive, then it could obscure the information females gain about male quality. But similar issues might affect any plastic condition‐dependent signal. In response, females might evolve mechanisms to adjust their evaluation of male quality based on the social context, similar to the enhanced selectivity of female field crickets (*Gryllus lineaticeps*) at higher male densities (Atwell & Wagner, 2014). Studies investigating analogous changes in female sensitivity to SP with the social context would be illuminating.

Second, the link between SP transfer and male quality is likely to be complicated by depletion through repeated mating. When males mate repeatedly, SP transfer declines with each mating, and levels are fully recovered with 3 days of sexual abstinence (Sirot *et al*., 2009). If high‐quality males achieve more matings than low‐quality males, then depleted high‐quality males might, counterintuitively, transfer less SP than low‐quality males. Similar reversed condition dependence occurs for ejaculate components in other animals (Preston *et al*., 2001; Perry & Rowe, 2010). However, even if high‐quality males mate more often, they might continue to transfer more SP compared with low‐quality males, either because they store more SP or because they replenish SP more rapidly, which will tend to restore quality dependence in SP transfer. An analogous process presumably underlies the condition dependence of male reproductive potential in some *Drosophila* species, in which only high‐quality males can maintain the transfer of very long, costly sperm over successive matings (Lüpold *et al*., 2016; see also Macartney *et al*., 2021). Even in the face of noisiness in the relationship between SP transfer and male quality, females might integrate information from multiple sources to assess male quality more accurately. For example, females might assess a male's recent sexual history based on cuticular hydrocarbon profiles (Yew *et al*., 2009; Everaerts *et al*., 2010), and update her assessment criteria accordingly to account for SP depletion. This possibility points to the need for studies that explore the plasticity of female post‐copulatory mate assessment.

The possibility that females assess male quality through SP transfer raises a new hypothesis about male mating strategies. Small males often mate less frequently than large males (Simmons, 1988; Sih, Lauer & Krupa, 2002; Perry, Sharpe & Rowe, 2009; Stiver & Alonzo, 2010; Wigby *et al*., 2016). This pattern is often viewed as a consequence of competition with larger males or female preference for larger males. But a lower mating rate might be an adaptive compensation. By mating less often, small, low‐quality males might be able to match the SP transfer of large, high‐quality males, especially if high‐quality males are SP‐depleted from frequent matings, an effect that would further weaken the strength of SP's condition dependence. Whether it really pays males to forgo mating opportunities to pursue fewer, higher investment matings (e.g. Macartney, Bonduriansky & Crean, 2020) will depend on the frequency of mating opportunities, the per‐mating benefit of transferring more SP, and the distribution of male quality within populations, and represents fertile ground for theory.

Overall, a full understanding of whether SP, or SFPs in general, function as signals of male condition requires experiments that determine how social context and repeated mating affect the link between male quality and transfer. The strength of this link will depend on male remating intervals in natural populations, how male remating and SP replenishment vary with male quality, and female ability to discriminate fine differences in SP titres.

##### (ii) Female discrimination between SP doses from multiple mates

After a first mating, a female can attribute the quantity of SP received to her mate. However, after several matings a female will retain SP from multiple males. Indeed, SP transferred by one male can bind to the sperm of a rival within the female storage organs (Misra & Wolfner, 2020). Thus, when ejaculates from different males overlap in a female's reproductive tract, the quantity of SP stored is not necessarily informative of the amount received from the last partner. In this case, to ascribe SP receipt accurately to a given partner, females would need to attend specifically to the amount received, rather than stored, or to the change in stored SP quantity pre‐ and post‐mating.

##### (iii) Evidence of seminal fluid toxicity evolving under polyandry

Several studies have evolved replicated populations of fruit flies under either strict monogamy, where the fitness interests of the sexes are aligned, or polygamy, where sexual conflict persists. These studies have not directly assayed evolved changes in SP transfer or female responses to SP. However, they have found that males evolving under polygamous mating have higher SFP expression (including SP), impose greater survival costs on their female partners, and induce greater stimulation of egg production and transcriptional responses in their partners (Holland & Rice, 1999; Hollis, Houle & Kawecki, 2016; Hollis *et al*., 2019) [for analogous results from experimental evolution with respect to sex ratio, see Wigby & Chapman (2004) and Nandy *et al*. (2013)]. These results suggest that some part of the male ejaculate evolves to be increasingly toxic to females under polygamous mating. However, an alternative explanation has not been refuted: males evolving under polygamy might evolve greater attractiveness, which conversely decays in monogamous populations (e.g. Debelle *et al*., 2017). Attractive males from polygamous conditions might provoke an extreme reproductive investment in non‐coevolved females, which manifests as an early‐life spike in reproduction and comes at a cost to longevity.

##### (iv) Why should females heed an ejaculate‐based signal?

SP is not the only condition‐dependent male phenotype in *D. melanogaster* (e.g. Bonduriansky *et al*., 2015). What information about males could SP provide, over and above these other traits? Firstly, there is growing evidence that female assessment of male signals continues after copulation (Firman *et al*., 2017), and the possibility that females assess male quality after copulation is consistent with a cross‐taxa trend towards strong condition dependence in seminal fluid quantity (Macartney *et al*., 2019). For SP in particular, an intriguing possibility is that its prolonged storage in female reproductive organs (~10–14 days; Peng *et al*., 2005a) gives a long‐term, physiological memory of SP transfer, generating a record of male quality or sperm transfer, or both. In an analogous way, selection is expected to act to enhance signal memorability for macroscopic signals (Guilford & Dawkins, 1991). Secondly, using multiple, redundant signals across different sensory modalities may increase the efficacy with which quality is advertised (Rowe, 1999) and ensure that responses are based on robust information (Dore *et al*., 2018).

## VI CONCLUSIONS

1. SP is a short protein, found in only a few *Drosophila* species, that is transferred to females by males during mating. Once inside females, it induces profound and remarkably varied changes. Its breadth of effects and limited phylogenetic distribution have rightly captured the interests of ethologists and evolutionary biologists for decades. It remains one of the best characterised reproductive proteins and represents a foundational case study in the fields of sexual selection, reproductive evolutionary biology, and sexual conflict.
2. SP has come to be widely viewed as an agent of manipulation used by males to alter female reproductive processes to the gain of the male transferring it and at the expense of the female receiving it. However, a purely antagonistic model for the evolution of SP is not well supported by the data. We have argued this from two perspectives. Firstly, in relation to data from contemporary populations of *D. melanogaster*, the weight of experimental studies suggests neutral or beneficial effects on female fitness, with net costs arising only under nutritional extremes. But the contexts investigated in laboratory studies are necessarily limited abstractions, and it remains to be seen how the directionality and magnitude of SP's fitness effects are influenced by the interacting forces of nutrition, disease, predation risk, encounter rate, climate, and, presumably, higher variance in male quality in wild‐living populations. We need studies that measure the fitness effects of SP under these conditions. Secondly, we argued in relation to the comparative functional and evolutionary genetics of the SP–SPR system. A pure sensory exploitation model for SP's evolution – based on SP exploiting pre‐existing SPR – is inconsistent with the more recent expansion of robust *SPR* expression into the female reproductive tract, the relationship between *MIP*‐ and *SPR‐*expressing neurons in controlling the post‐mating response, the highly restricted localisation of SP‐sensing neurons in the reproductive tract, and the independence with which changes in SPR sequence can affect SP‐ and MIP‐binding activity. We conclude that the evolutionary order of events in the SP–SPR system appears to suggest the evolution of a dedicated SP‐sensing system, which presumably evolved through female benefit.
3. There are two principal and non‐mutually exclusive ways in which females might benefit from sensing SP. Firstly, they could use the receipt of SP, perhaps ancestrally transferred as a structural component of seminal microcarriers, as a cue of mating. Responding to this cue would enable the tight coordination of reproductive processes and mating decisions with sperm receipt. The evolution of SP‐binding to sperm would further allow for coordination with sperm storage. Secondly, quantitative (or qualitative) variation in SP transfer might covary with male condition. Females could use this information about male condition to guide their reproductive investment decisions (differential allocation or selectivity when remating) in order to capitalise on the good gene (or direct) benefits of mating with high‐quality males. There is some evidence that SP meets the criteria for condition‐dependent signals: females can benefit from SP receipt, SP transfer covaries with male condition, and females appear to tailor some parts of their reproductive effort to the amount of SP they receive. However, the data are inconclusive and more study is required. We need to be able to distinguish the effects of SP from the myriad other ejaculate components, in terms of both the degree of condition dependence it exhibits and its specific contribution to quantitative variation in female post‐mating responses.
4. A narrow focus on sexual conflict over SP's effects on females neglects more expansive frameworks that recognise the contribution of both antagonistic and cooperative processes to the evolution of SP (and reproductive traits more broadly). SP's effects on females may have originated as a cue, a signal, or through sensory exploitation; subsequently evolved *via* sexually antagonistic coevolution or mutually beneficial ritualisation; and even transitioned from manipulation to cooperation or *vice versa*. As long as male and female interests are not perfectly aligned, conflict will inevitably persist, perhaps manifesting as continued low‐level exploitation within a broadly honest, established signalling system (Krakauer & Johnstone, 1995). In this sense, juxtaposing ‘conflict’ *versus* ‘signalling’ explanations for the evolution of SP (and any sexual trait) represents a false dichotomy: both processes can have made different contributions at different points in SP's evolutionary history, their relative influence shifting through time and the fitness effects of SP changing with the ecological context.
5. A more expansive view that considers these different forces illuminates questions that need answers to understand SP's evolution. Did the evolution of *SPR* expression in the female reproductive tract allow SP detection for the first time, or did it enhance a pre‐existing female ability to detect SP? If SP transfer is condition dependent, was it always so or did condition dependence evolve following female ability to detect SP within the reproductive tract? Did the ability of SP to bind sperm and therefore remain detectable over prolonged periods pre‐date female ability to detect SP in the reproductive tract? Which party controls the cleavage of SP from the surface of sperm? Answering these questions will help to resolve the evolutionary assembly of the SP–SPR system, a focal point for understanding the evolution of reproductive interactions.
6. Our discussion of the conditions under which SP could function as a signal, either of male quality or of mating and sperm storage, represents an approach to treating molecular signals as animal signals. Molecular signals raise new questions about signal evolution. For one, a molecular signal like SP does not act through the receiver sensory modalities (olfaction, sound, vision) traditionally associated with signals; interestingly, the SP‐sensing neurons in the female reproductive tract share transcriptomic similarities with proprioceptive neurons (Rezával *et al*., 2012). How does this difference in signal modality affect evolutionary change in signal structure? Is the neural circuitry used to process macroscopic signals more susceptible to cognitive biases and illusions (Guilford & Dawkins, 1991; Kelley & Kelley, 2014) than those used to process molecular signals? Are molecular signals more or less constrained in their evolution than their macroscopic counterparts? How can the honesty of a condition‐dependent signal be maintained in the face of molecular depletion? Along with raising new questions, molecular signals provide new, powerful opportunities for testing long‐standing predictions in ways that would be challenging for macroscopic signals. These include testing whether the rate of evolutionary change differs between the primary signal and its amplifiers (in SP's case, the cofactor network upon which SP function depends) and inducing the upregulated expression of a molecular signal in low‐quality males to test for the predicted costs of signal dishonesty (Biernaskie, Grafen & Perry, 2014). We see molecular signalling systems, such as SP–SPR, as uniquely tractable systems for unravelling general principles guiding the evolution of animal signalling systems. The (relative) ease of phenotypic engineering, experimental evolution, and detailed genetic manipulation in *Drosophila* offer huge promise for establishing SP as a textbook animal signalling study system.
7. While the SP–SPR system appears confined to a subset of drosophilids, a substantial increase in egg‐laying and a reduction in sexual receptivity following receipt of accessory gland products are widespread phenomena among insect species (reviewed in Chen, 1984; Gillott, 2003; Hopkins, Avila & Wolfner, 2018). In a handful of cases, the specific products driving these changes have been identified, including receptivity‐ and calling‐inhibiting substance (RCIS) in the moth *Helicoverpa armigera* (Kiran *et al*., 2021), pheromonostatic peptide (PSP) in *Helicoverpa zea* (Kingan, Thomas‐Laemont & Raina, 1993), and head peptide‐I (HP‐I) in *Aedes aegypti* (Duvall *et al*., 2017). The SP–SPR system we have discussed in this review may, at a mechanistic level, be a *Drosophila* quirk. But the fundamental relationship between male seminal fluid and the female post‐mating response is apparently ubiquitous among insects. These observations raise two fundamental questions: why is male influence over female post‐mating behaviour so common? And why are there so many lineage‐specific molecules by which males influence it? Given the ever‐increasing depth with which the system is understood, the study of SP in *Drosophila* may be our most powerful tool for answering these questions.
